# Exploring the clinical value of concept-based AI explanations in gastrointestinal disease detection

**DOI:** 10.1038/s41598-025-14408-y

**Published:** 2025-08-07

**Authors:** Andrea M. Storås, Maximilian Dreyer, Frederik Pahde, Sebastian Lapuschkin, Wojciech Samek, Pål Halvorsen, Thomas de Lange, Yuichi Mori, Alexander Hann, Tyler M. Berzin, Sravanthi Parasa, Michael A. Riegler

**Affiliations:** 1 Department of Holistic Systems, SimulaMet, Oslo, Norway; 2 Department of Computer Science, OsloMet - Oslo Metropolitan University, Oslo, Norway; 3 Explainable Artificial Intelligence Group, Fraunhofer Heinrich Hertz Institute, Berlin, Germany; 4 Department of Electrical Engineering and Computer Science, Technical University of Berlin, Berlin, Germany; 5 BIFOLD - Berlin Institute for the Foundations of Learning and Data, Berlin, Germany; 6 Department of Medicine and Emergencies - Mölndal, Sahlgrenska University Hospital, Region Västra Götaland, Gothenburg, Sweden; 7 Department of Molecular and Clinical Medicine, University of Gothenburg, Gothenburg, Sweden; 8 Clinical Effectiveness Research Group, University of Oslo and Oslo University Hospital, Oslo, Norway; 9 Gastroenterology section, Department of Transplantation Medicine, Oslo University Hospital, Oslo, Norway; 10 Digestive Disease Center, Showa University Northern Yokohama Hospital, Yokohama, Japan; 11 Interventional and Experimental Endoscopy (InExEn), Department of Internal Medicine II, University Hospital Würzburg, Würzburg, Germany; 12 Center for Advanced Endoscopy, Beth Israel Deaconess Medical Center and Harvard Medical School, Boston, Massachusetts United States; 13 Department of Gastroenterology, Providence Swedish, Seattle, Washington United States

**Keywords:** Explainable artificial intelligence, Concept explanations, Deep learning, Gastroenterology, Gastrointestinal diseases, Machine learning, Computer science, Medical imaging

## Abstract

Complex artificial intelligence models, like deep neural networks, have shown exceptional capabilities to detect early-stage polyps and tumors in the gastrointestinal tract. These technologies are already beginning to assist gastroenterologists in the endoscopy suite. To understand how these complex models work and their limitations, model explanations can be useful. Moreover, medical doctors specialized in gastroenterology can provide valuable feedback on the model explanations. This study explores three different explainable artificial intelligence methods for explaining a deep neural network detecting gastrointestinal abnormalities. The model explanations are presented to gastroenterologists. Furthermore, the clinical applicability of the explanation methods from the healthcare personnel’s perspective is discussed. Our findings indicate that the explanation methods are not meeting the requirements for clinical use, but that they can provide valuable information to researchers and model developers. Higher quality datasets and careful considerations regarding how the explanations are presented might lead to solutions that are more welcome in the clinic.

## Introduction

Performing endoscopies of high quality is important for proper examination of the gastrointestinal (GI) tract. The quality of the procedures can be measured by key performance indicators ^1^. Examples include caecal intubation rate and rectal retroflection to ensure that the entire bowel is examined, the adenoma detection rate to prevent development of cancer, and bowel preparation to describe how clean the bowel is during examination ^2^. Early detection of polyps and tumors in the GI tract has shown to improve clinical prognosis and reduce cancer incidence and associated mortality ^3–5^. Automatic analysis of videos and images from the GI tract using deep neural networks (DNNs) can speed up interpretation and might reduce the miss rate for abnormal findings. Since DNNs are highly complex and challenging to interpret, explainable artificial intelligence (XAI) methods are useful to provide a deeper understanding of how these models work and most interestingly where they fail and why. Earlier work has shown that machine learning (ML) models, including DNNs, can learn spurious correlations in the data, reducing their analytic capabilities. This is also true in the medical domain ^6–8^. To avoid implementing sub-optimal ML systems in the clinic, it is important for developers to discover and be aware of such limitations. Furthermore, ensuring that ML systems are transparent and well understood by the users is crucial in medicine, where high-stakes decisions are made. Consequently, healthcare personnel should be consulted when deciding how to explain DNNs for medical applications.

**Fig. 1:**
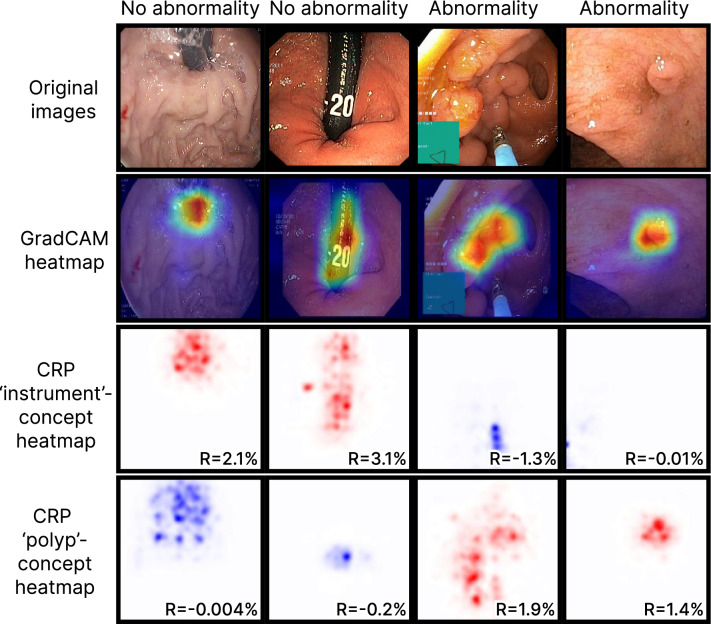
Original images and corresponding heatmap explanations using GradCAM and CRP. For each CRP explanation, we report the importance (R) of the respective concept (‘instrument’ or ‘polyp’) for the prediction outcome.

XAI is a subsection of artificial intelligence (AI) that includes methods for explaining ML models and their predictions. As the importance of understanding ML models applied in critical fields like medicine has become widely recognized, the number of XAI methods has increased , see for example ^9^ . Some approaches that have been applied for medical used cases include SHapley Additive exPlanations (SHAP) ^10^ , DeepXplainer ^11^ , $\textrm{BC}^2$ ^12^ and AC2 ^13^ . One popular group of methods for explaining DNNs that analyze images are XAI methods producing heatmaps. These methods present the model explanation for a specific prediction to the user in form of a heatmap, highlighting the regions in the image that are most important for the model prediction. Examples of heatmaps are shown in the second row of Fig. 1. Presenting the explanation visually can make the heatmaps intuitive to interpret which, at least partially, explains their high popularity for image analysis. A popular method producing heatmaps called GradCAM ^14^ was recently evaluated by medical experts to explain a DNN detecting polyps in the GI ^15^. The results show that these heatmaps were preferred above heatmaps produced by the SHAP explanation method ^10^.

**Fig. 2:**
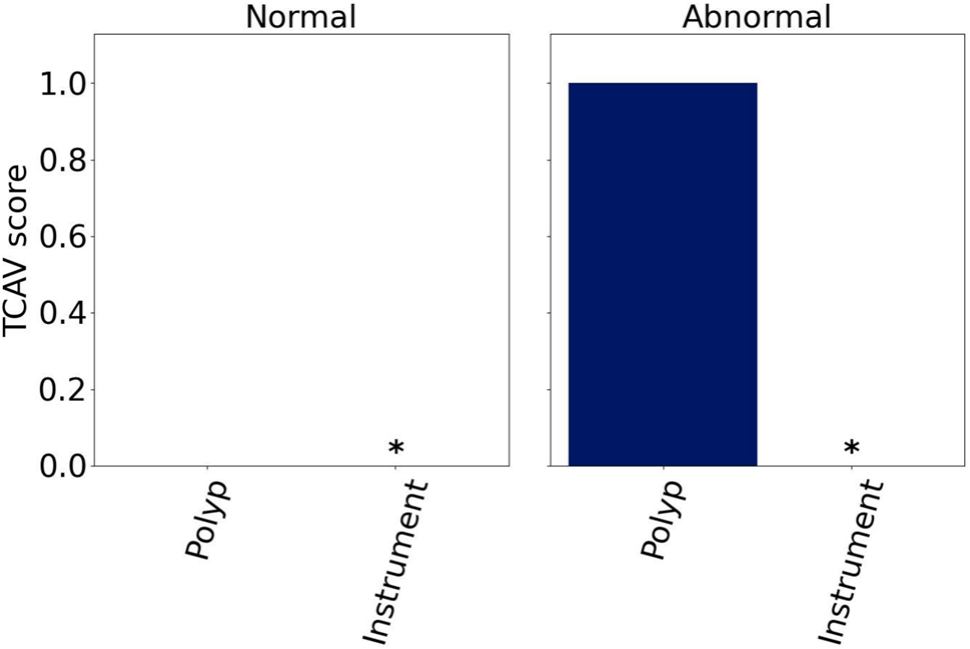
Mean TCAV scores for the ‘polyp’ and ‘instrument’ concepts for representative ‘normal’ and ‘abnormal’ images. $*$ marks insignificant concepts, and their corresponding scores are therefore not shown.

Despite their popularity, traditional heatmaps only indicate *where* in the image the model focuses, but not *what* that image region represents. Consequently, it can be unclear why the model pays attention to the highlighted area, and it is left to the user to interpret what the model reacts to. Furthermore, the methods produce one heatmap per individual model prediction, meaning that it becomes challenging to explain the global model behaviour. Concept-based XAI methods aim to tackle some of these limitations by generating explanations that build on human-understandable concepts relevant for the specific use case. Here, we describe a concept as a higher-level topic or theme, such as ‘stripes’ or ‘dots’. The high flexibility regarding which concepts to explore makes it easy to tailor the explanations to the task of interest, such as the interpretation of medical images in the GI tract. Several concept-based methods exist. This work explores two of them, namely Testing with Concept Activation Vectors (TCAV) ^16^ and Concept Relevance Propagation (CRP) ^17^. TCAV shows the relative importance of different concepts for the DNN when analyzing a group of images ^16^. The concepts are defined by the user by selecting representative images. Consequently, the resulting explanations can be tailored to the specific task. Figure 2 plots TCAV scores for two different image classes. The CRP framework explores concepts that are intrinsically learned by the model, meaning that the concepts are not defined beforehand by the user ^17^. Unlike TCAV, CRP generates concept-specific heatmaps that localize the concept in the input image. This is illustrated in the two bottom rows of Fig. 1, where each row corresponds to one concept. More details about the methods are provided in the Methods section and in the respective original publications.

This article explores three XAI methods for gaining insights into a DNN detecting abnormalities in images from the GI tract. GradCAM ^14^ and the concept-based methods TCAV ^16^ are selected due to their promising results in medical imaging ^15,18^ . CRP ^17^ has several advantages above TCAV that makes it interesting to investigate. To our knowledge, this is the first time these XAI methods are compared for explaining a DNN analyzing GI images. Rather than relying on quantitative evaluation metrics, we collect qualitative feedback on the model explanations from gastroenterologists to assess the utility of the methods from a medical perspective. Overall, their preferences vary regarding which explanations to receive in a clinical setting, underscoring that human interaction with ML systems is subjective. The model learned concepts related to medical findings, conditions and anatomical landmarks. The quality of the images used for training and explaining the model will affect the quality of the model explanations.

## Results

This section first reports the performance of the DNN in classifying images as ‘abnormal’ or ‘normal.’ Next, the model explanations generated using GradCAM, TCAV and the CRP framework are presented. Finally, the feedback on the explanations from experienced gastroenterologists is provided.

### Model performance

The DNN was based on a pretrained ResNet152 ^19^ and fine-tuned on images from Hyperkvasir ^20^ and ImageNet ^21^ as described in Methods. The accuracy on the hold-out test set was 0.953, and the precision, recall and Matthew’s correlation coefficient (MCC) were 0.951, 0.949 and 0.900, respectively. The accuracy on the representative external test set was 0.880, and the precision, recall and MCC were 0.886, 0.880 and 0.766. Because the representative test set included images from other datasets than the training and validation data, observing a slight drop in model performance is expected. While model performance directly reflects a model’s abilities to solve a specific task, the performance is also relevant from an explainability perspective. If the model cannot successfully classify observations, the corresponding model explanations are less likely to highlight task-related features. The performance metrics above indicate that our model is capable of separating images with and without abnormal findings, respectively.

### GradCAM explanations

A selection of heatmaps from the last convolutional layer in the model is included in the second top row of Fig. 1. Corresponding original images provided to the model are shown in the top row. The two columns to the left side of the figure show results for ‘normal’ images, while the two columns to the right show results for ‘abnormal’ images. Red and blue colors indicate the areas that are most and least important for the model prediction.

### TCAV explanations

Figure 2 plots the mean TCAV scores across the 20 pairs of positive and negative example sets for each combination of concepts and images classes. The scores are calculated for the representative test set. Only the polyps concept was significant. For the ‘abnormal’ class, the TCAV score was 1 for all the pairs of example sets. This is expected because all the abnormal images in the representative test set included polyps. The TCAV score was 0 for the ‘normal’ class, where none of the images contained polyps. Because instruments were present in both categories of diseases, it is not surprising from a medical perspective that the instrument concept was insignificant for the model when distinguishing between ‘abnormal’ and ‘normal’ images.

### CRP explanations

CRP heatmaps for the concept channels with the highest mean values in the concept activation vectors (CAVs) are shown in the lower part of Fig. 1. Red color indicates positive relevance for the final model prediction, and blue color indicates negative relevance. The second bottom row in the figure includes CRP heatmaps for the ‘instrument’ concept, while the bottom row provides ‘polyp’ concept heatmaps. Different concept channels were identified for the two image classes. For the ‘instrument’ concept and ‘normal’ images, channel 1063 was applied. Channel 682 was used for the ‘abnormal’ images. Channels 114 and 421 were used for generating the ‘polyp’ concept heatmaps for ‘normal’ and ‘abnormal’ images. From the figure, we observe that the ‘instrument’ and ‘polyp’ heatmaps tend to highlight areas in the image that include instruments and polyps, respectively. The relevance score measures how much the output from a channel contributes to the final model prediction. Relevance scores are embedded in the CRP heatmaps in Fig. 1

**Fig. 3:**
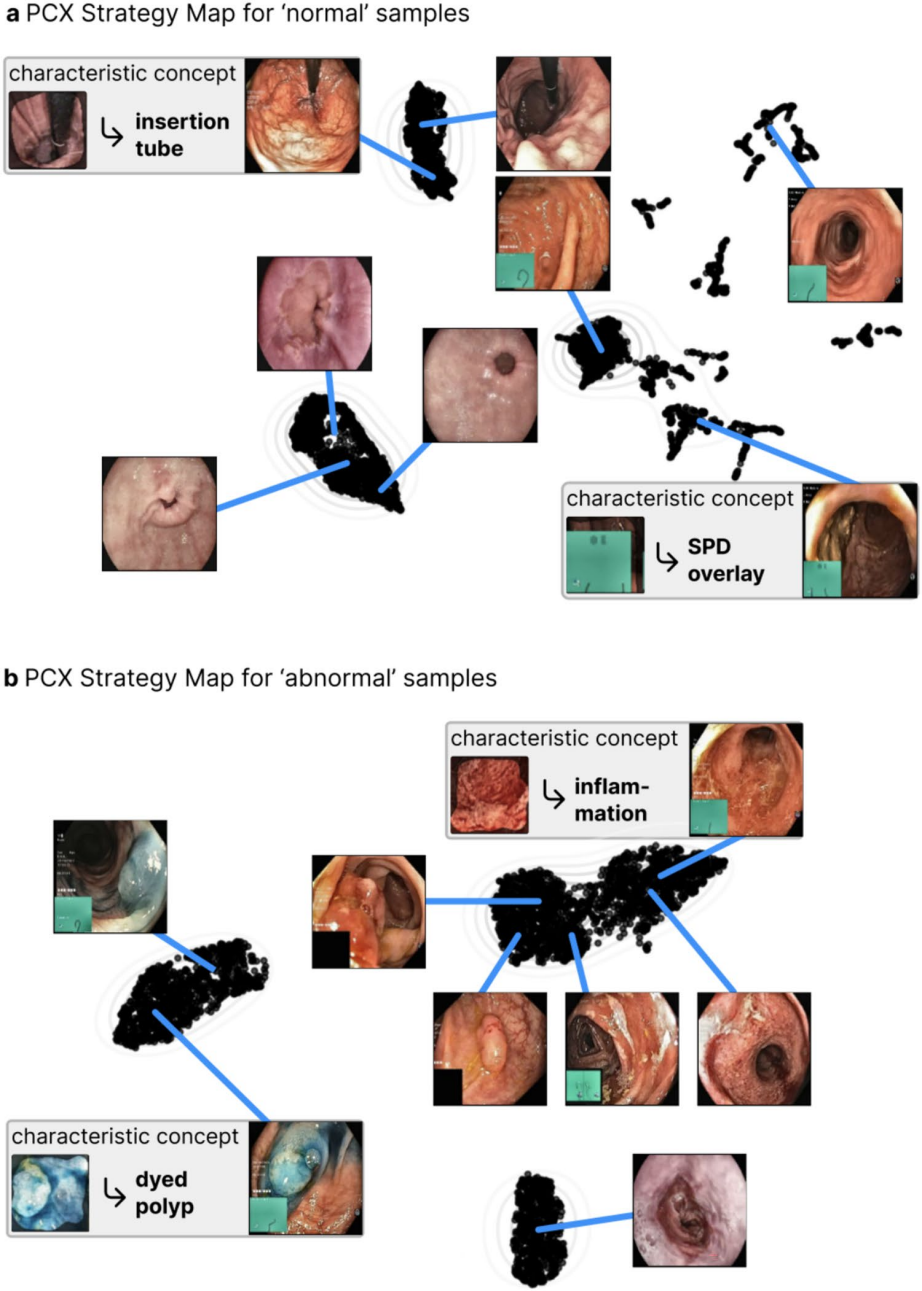
Understanding the global model behavior with concept-based prototypes using PCX for **a**) normal and **b**) abnormal samples. The PCX Strategy Maps show distinctly forming clusters of explanations, with eight prototypical examples shown (four for each image class). For each cluster, characteristic concepts are important. For the normal samples, PCX reveals that, e.g., the insertion tube or green picture-in-picture of a scope position device (SPD) is learned and used by the model for its decision-making. Regarding the abnormal samples, characteristic concepts for inflammation (right hand side of the cluster to the upper right) and dyed polyps are found.

The Prototypical Concept-based Explanations (PCX) framework clusters latent concept relevances per class to identify prediction substrategies applied by the model, that can be represented by prototypical samples ^22^. Figure 3 shows the prototype maps for ‘abnormal’ and ‘normal’ images in the test dataset from Hyperkvasir when using PCX. Points that are close to each other in the map, correspond to similar predictions (and explanations). The most representative image for each prototype are shown in the figure. Importantly, PCX allows understanding the concepts that are characteristic for each prototype, revealing if the model relies on spurious and/or expected features. Detailed PCX explanations with all characteristic concepts are given in Supplementary A.

### Expert feedback on model explanations

All five gastroenterologists responded to the questionnaire. They were all highly experienced endoscopists and had knowledge about ML. Table 1 summarises the overall impression of the model explanations for each participant.

**Table 1:** The study participants’ overall impression of the explanation methods, categorised as being positive, negative or mixed. Abbreviation: P: participant.

**Explanation method**	**P1**	**P2**	**P3**	**P4**	**P5**
GradCAM heatmaps	Positive	Positive	Mixed	Negative	Positive
CRP heatmaps	Negative	Negative	Positive	Negative	Positive
TCAV scores	Positive	Positive	Negative	Negative	Positive
PCX visualizations	Positive	Positive	Negative	Positive for model development	Positive
Prototype-concept matrices	Positive	Positive	Negative	Positive for model development	Positive
Preferred explanation type	GradCAM	Prototype-concept matrices	GradCAM or CRP (depending on use)	No explanations	Combination of GradCAM, CRP and TCAV

Four out of five participants were positive toward receiving model explanations. The heatmaps produced by GradCAM were regarded as easy to interpret. The CRP heatmaps were found to include more information. Several participants stated that this could be a barrier for clinical use. It was repeatedly mentioned that the information provided should be as simple as possible. One participant, on the contrary, appreciated the additional information captured by the CRP heatmaps. The participant found the combination of several explanation methods useful for clinical practitioners because the explanations complement each other and provide increased insights into how the model analyses images. The PCX prototype maps and prototype-concept matrices were not regarded as intuitive to interpret. In addition, some found the representative images too small for detailed examination. Regarding the PCX prototype map for ‘abnormal’ images, a distinct cluster of images representing blue dyed polyps was observed to the left.

Interestingly, the majority of prototypes identified by the model could be recognized as specific medical findings, conditions or anatomical landmarks. Different prototypes sometimes represented the same medical pattern, e.g., two prototypes represented polyps in the colon. The participants largely agreed on what the prototypes in the prototype-concept matrices and PCX prototype maps represented. However, the small size of the example images in the survey made them difficult to study in detail. This practical challenge might contribute to some of the deviations in response between gastroenterologists.

The TCAV score plots were overall well received, and the resulting scores seemed to align with the expectations for the current use case. While one participant highlighted that the plots were good for summarizing multiple values, another participant found the amount of information to be too sparse. A third participant was uncertain how the accuracy of the TCAV scores would change if more concepts were included. Finally, one participant thought the TCAV scores were useful to assess whether the model pays attention to the ‘right’ patterns in the data, which is crucial for clinical acceptance.

Regarding whether a combination of explanations could be valuable, the majority of the participants agreed that only one explanation type should be provided to keep the information as simple as possible. However, there was no unison agreement on which explanation method to prefer. GradCAM heatmaps were perceived as easy to interpret, making them the preferred choice by one participant. Another participant stated that the choice of explanation depended on what he would like to study. For detecting findings, GradCAM heatmaps were preferred, while CRP heatmaps were regarded as more appropriate for characterizing findings. The third participant preferred the concept explanations from CRP along with images that are most representative for each concept. As opposed to the three first participants, one participant would like to receive GradCAM and CRP heatmaps and TCAV scores. The different explanation methods contribute to different insights into the ML model, which was regarded as valuable during diagnostic decision-making and ongoing model development. Finally, one participant was reluctant to receiving model explanations at all during clinical procedures. According to him, the explanations were likely to be more useful for scientists than clinicians.

## Discussion

This study focuses on qualitatively evaluating the clinical usefulness of a selection of XAI methods. Feedback from gastroenterologists shows that their XAI preferences vary: Not all prefer receiving model explanations in the clinic, and their XAI methods of choice differ. Because the human interaction with ML models is highly subjective, future research may focus on XAI tools tailored to each practitioner’s preferences. Ensuring streamlined interaction with the medical ML system for all of its diverse users is crucial not only to increase the acceptance ML tools, but also to contribute optimizing the workflow and handling patients faster.

Table 2 summarizes the pros and cons for the explanation types explored in this study, also considering the response from the medical experts. Even though GradCAM heatmaps were regarded as intuitive by the study participants, these heatmaps are not necessarily straightforward to interpret. One medical doctor found the heatmaps useful to investigate whether the clinicians and models focus on the same area in the image. However, as reflected upon by another participant, this way of evaluating model explanations could actually make the explanations less useful in clinical practice. The reason is that ML models do not necessarily interpret images in the same way as medical doctors. Consequently, if the explanation shows that the model pays attention to other details than what the medical doctor does, it could reduce the doctor’s trust in the model even though the model actually is correct. In such a situation, the explanation would be disturbing rather than helpful. The potential issue arising from discrepancy between areas highlighted in the heatmaps compared to the medical doctors’ ground truth areas was also reflected in the comments from one of our study’s participants. This concerned both GradCAM and CRP heatmaps.

**Table 2:** Overview of pros and cons for the explanation types explored in this work, taking the feedback from experienced gastroenterologists into account.

Explanation type	Pros	Cons
GradCAM heatmaps	Intuitive to interpret	Disagreement between highlighted area in heatmap and clinicians’ expectations can reduce model trust
	Not containing too much information	Can only explain a single prediction at a time
	Easy to implement	No information about what the highlighted areas represent
CRP heatmaps	Information about what the highlighted areas represent	Complex explanations because one heatmap is generated for each concept
	Easy to implement with open-source frameworks	Can only explain a single prediction at a time
TCAV scores	Not containing too much information	Too little information in the explanations
	Can explain overall model behaviour for a group of observations	Must specify concept example images for implementation
	Can assess whether the model focuses on relevant patterns	
PCX prototype maps	Can explain overall model behaviour for a group of observations	Less intuitive to interpret
	Enables identification of ways to improve the model	Not preferred for clinical use
Prototype-concept matrices	Can explain overall model behaviour for a group of observations	Less intuitive to interpret
	Can assess whether the model focuses on relevant patterns	Not preferred for clinical use

According to the gastroenterologists, CRP heatmaps were regarded as more complex to interpret than GradCAM heatmaps. This was expressed as a potential limitation for using CRP heatmaps in the clinic, where explanations should be intuitive and fast to interpret. From a medical perspective, it could be relevant to study more concepts in addition to ‘polyps’ and ‘instruments’. However, this would lead to even more complex model explanations that can be more distracting than supportive during a clinical procedure. Another concern was that the clinical ‘correctness’ of the explanations might decrease when adding more concepts. By improving the interface, CRP heatmaps might become more applicable in the clinic. It was suggested that combining different concept heatmaps for the same predicted image into a single heatmap where different colors represent different concepts could be useful. Defining more specialized concepts, e.g., concepts describing the structural surface of polyps, and combining the heatmaps might lead to faster polyp characterization.

The study participants recognized the potential for the CRP-based explanations during model development. As an example, the prototype-concept matrices can identify outlier images and suggest if more training data should be collected for specific disease classes. When inspecting the PCX prototype map for ‘abnormal’ images, the participants observed that the dyed polyps are clearly separated from other polyps. One participant pointed out that computer-based tools for interpreting GI images typically struggle when the appearance of the polyp changes during a procedure, making the computer program interpret the same polyp as being several different polyps. Based on our PCX prototype map for ‘abnormal’ images where blue dyed polyps are clearly separated from other polyps, this seems to be an issue for our DNN as well. In other words, we find that the CRP-based explanations can detect limitations that could negatively affect the model performance on new images.

For the CRP heatmaps of the ‘instrument’ concept, the ‘normal’ images to the left in Fig. 1 received positive relevance scores, suggesting that the model associates instruments with the ‘normal’ class. Because an instrument is not a sign of disease absence, this could be a spurious correlation learned by the model. To investigate the finding further, we cropped instruments (insertion tubes) from images with instruments and inserted them in images without instruments, as proposed in^7^. More details are given in Supplementary B. When analyzing the manipulated images, the model accuracy dropped significantly for the ‘abnormal’ class (from 0.943 to 0.783) while the accuracy increased for the ‘normal’ class (from 0.949 to 0.967). Additionally, images with different instrument types were explained using CRP and the most relevant concepts were inspected. The results are included in Supplementary C. Except for images containing bright white instruments, the most relevant concepts represented an instrument (Figure C.1). For bright white instruments, the most relevant concepts did not represent instruments (Figure C.2). Interestingly, instruments marked with the number 20 were associated with the ‘abnormal’ class (Figure C.3). Another concept representing black edges and text was found highly relevant for the ‘abnormal’ class.

As opposed to the CRP explanations, the TCAV scores show that the ‘instrument’ concept is insignificant for the ‘normal’ class. The divergent results are likely caused by the different logics behind CRP and TCAV. First, concept relevance scores are based on a single channel, or neuron , for the layer in the DNN that is explored, assuming that one channel represents one concept. TCAV, on the other hand, is based on a vector (CAV) for the concept , corresponding to a linear combination of all the neurons in the investigated layer, and checks how sensitive a model prediction is to changes in the direction of the CAV ^16^. Our experiment on manipulating images by inserting instruments indeed showed that the model is sensitive to such changes. In other words, TCAV shows limitations at identifying the high importance of concepts compared to CRP. Recent work ^23^ has pointed out that the limitations of TCAV in faithfully attributing concepts can result from incomplete or skewed estimates of the concept direction, especially when relying on linear classifiers. As such, it can be beneficial to apply complementary XAI methods like CRP. Notably, however, CRP is only applicable when concepts are encoded by single neurons . Taken together, even though there are some fundamental differences in how TCAV and CRP create model explanations, their explanations only disagree for the importance of the ‘instrument’ concept for the ‘abnormal’ class.

Our study has some limitations. First, the quality of images used to train, evaluate and explain the DNN are not optimal compared to the newest technology available on some hospital wards. Obtaining high-resolution images might improve model performance ^24^. Moreover, it could contribute to more fine-grained annotations. Such annotations open up to increase the number of possible diagnoses predicted by DNNs. They also allow for more detailed concept explanations that, for example, can help clinicians characterising the surface of polyps. The second limitation of this work is the limited number of concepts that were explored. Although this study is the first of its kind in the GI domain, future work should spend more effort expanding the number of concepts. To ensure clinical relevance of additional concepts, concepts should be selected in collaboration with gastroenterologists. It is also relevant looking into even more concrete tasks in medical practice in the context of GI disease detection and collecting corresponding input from healthcare personnel. A third limitation is the absence of quantitative performance metrics for the model explanations. The primary focus of this pilot study was to evaluate the clinical interpretability and utility of the explanation methods. For clinical adoption of the explanation methods, qualitative feedback from domain experts was viewed as more directly relevant than quantitative metrics for the resulting model explanations. However, such metrics would be valuable to the field. Consequently, future work should develop standardized quantitative evaluation metrics for concept-based explanation methods in medical imaging, including the creation of annotated datasets for this purpose.

We experience that not all clinicians find it necessary or useful to receive model explanations in a clinical setting. Because medical doctors are busy and often need to make decisions fast, receiving too much information can become a hinder rather than making them more efficient. As long as the underlying model works for the clinically ‘correct’ reasons, the explanations can be distractive. It could be argued that it is more important to receive model explanations during the model development stage in order to identify bugs and weaknesses ^25^. Gaining increased understanding about how complex DNNs analyze medical data is also valuable in research because it might lead to new medical knowledge.

The expectations and needs for model explanations are highly dependent on the human users that will receive the explanations. Therefore, the medical doctors’ feedback in the current study is expected to be affected by the doctors’ prior medical experience. Because the participants are diverse in terms on geographic locations and work place, we do not believe the feedback to be highly biased. Before implementing explanation methods, we strongly recommend to map who will be the receivers of the explanations and in what kind of setting they will be. If we aim to apply explanations in the clinic, we should be considerate about how this can be accomplished in a simple manner that does not distract the clinicians from doing their work.

To conclude, complex AI models have potential to relieve clinicians of some of their tasks, enabling them to focus on more challenging medical work where human interaction is essential. However, a lack of understanding of how the models work and their limitations is likely to hamper the implementation of this technology in clinical practice. We explore several promising tools to explain a DNN trained to detect diseases in GI images. Feedback from experienced gastroenterologists indicates that the explanations are not fully ready to provide useful information to medical professionals in the clinic. However, the explanations can provide important insights to model developers and researchers. Higher quality datasets , quantitative performance metrics for concept-based explanations and careful considerations regarding how explanations are presented might lead to solutions that are more welcome and useful in the clinic.

## Methods

We develop a DNN to identify GI images containing abnormal findings, i.e., images showing a disease in the GI tract. The model is trained on images from the labeled subset of the Hyperkvasir dataset ^20^. Images representing barretts, esophagitis, polyps, ulcerative colitis or hemorrohoids are defined as ‘abnormal’, while images of anatomical landmarks in the GI tract (pylorus, retroflex-stomach, z-line, cecum, ileum and retroflex-rectum) as well as quality of mucosal view are defined as ‘normal’. All experiments are carried out in accordance with relevant guidelines and regulations. The medical datasets applied in the current work are fully anonymized, and the data collection has been approved by the Norwegian Privacy Data Protection Authority ^20^.

### Model

A ResNet152 ^19^ model that was pretrained on ImageNet ^21^ is fine-tuned for 200 epochs on GI images, and the weights from the version performing best on the validation set are saved as the final model. Due to class imbalance with the majority of images belonging to the ‘normal’ class, class weights are applied during fine-tuning. Similar to the original Hyperkvasir dataset experiments, a stochastic gradient decent (SGD) optimizer with learning rate 0.001 is applied. The CrossEntropyLoss from PyTorch is used as criterion ^26^. The final model prediction layer is modified to include two outputs: one output for each class. Moreover, identity layers are added after layer1, layer2, layer3 and layer4. Identity layers are placeholder layers allowing the explainability methods to investigate the features directly after the skip connections in the ResNet152 model architecture.

### Data

9542 ‘abnormal’ and ‘normal’ images from Hyperkvasir ^20^ are split into $70 \%$ for training, $20 \%$ for validation and $10 \%$ for testing. Earlier research has underlined that medical images and natural images tend to be analyzed differently by DNNs ^27^. Because we apply a model that is pretrained on ImageNet images, which includes natural images, the model might include latent features that affect its predictions on the medical images. Consequently, randomly selected images from ImageNet ^21^ are added to the ‘normal’ class for the train, validation and test sets. For the ‘normal’ class in the training set, 700 images are added. 200 and 100 images are added to the same class in the validation and test sets, respectively. The final model is evaluated on the test set with and without ImageNet images in the ‘normal’ class. Additionally, the model is evaluated on an external representative test set including images from the Kvasir-instrument ^28^ and Kvasir-SEG ^29^ datasets. All data sets are publicly available, and an overview of the data used for model development and evaluation is provided in Table 3.

**Table 3:** Origin and number of of images used in the current work reported as ‘normal’/‘abnormal’. *The model is also evaluated on the test set without Imagenet images. Abbreviation: NA: not applicable.

Dataset type	Hyperkvasir	Imagenet	Kvasir-instrument	Kvasir-SEG	Total
Train	4128/2550	700/0	NA	NA	4828/2550
Validation	1179/728	200/0	NA	NA	1379/728
Test*	591/366	100/0	NA	NA	691/366
Representative test	NA	NA	50/20	0/30	50/50

Preliminary experiments indicated that the model reacted to spurious patterns in the GI images like black corners and bright reflections. Consequently, we added image augmentation techniques. To remove areas that include the black edges, training, validation and test images are cropped before they are provided to the model. Next, Contrast Limited Adapted Histogram Equalization (CLAHE) ^30^ is applied to handle potential issues with bright reflections . The CLAHE implementation from the albumentation library is used, setting the clip_limit parameter to 2 ^31^.

### Model explanations

The resulting model is explained using traditional heatmaps from GradCAM ^14^ and by applying two different concept-based methods: TCAV ^16^ and CRP ^17^. No generative modeling is used. All explanations are given for images in the hold-out test set. For the GI use case, we explore polyps and instruments as concepts. Since polyps are included in some, but not all, images representing the abnormal class, we expect the model to pay attention to this concept when detecting abnormal images. Instruments are present in images from both classes and are expected to be less important for the model when learning to separate images with and without abnormalities. For all explanation methods, the last convolutional layer, i.e., layer 4, is investigated. This layer includes 2048 channels. Below are some details of the implementation for GradCAM, TCAV and CRP. The source code is publicly available: https://github.com/AndreaStoraas/conceptXAI-GItract.

#### GradCAM explanations

GradCAM is a gradient-based explanation method. Briefly, it takes the *gradient* for a specific model prediction. The gradient quantifies how much the output of the model changes when the input image is slightly changed. The gradients are multiplied with the activations for the layer of interest, making the heatmaps having the same resolution as the layer in the model that is explained ^14^. For creating the GradCAM heatmaps, the implementation in Pytorch by Gildenblat et. al.^32^ is used.

#### TCAV explanations

Concept images that represent polyps and instruments are selected for obtaining the concept activation vectors with TCAV. These concept images are taken from the Hyperkvasir ^20^, Kvasir-instrument ^28^ and Kvasir-SEG ^29^ datasets. Concept images can be part of the training data. 20 pairs of positive and negative example sets are created for both concepts. Each example set includes 45 randomly selected images that are manually inspected to ensure relevant content . To avoid issues related to overlap between the two concepts, relative CAV are obtained by following the original TCAV paper ^16^: For a given concept, the presence of other concepts should be balanced between the negative and positive example images. In our work, 20 of the positive and negative example images for the polyp concept include instruments, while the same number of example images for the instrument concept includes polyps.

#### CRP explanations

The CRP framework assumes that individual channels in a DNN represent distinct concepts. While CRP can identify concepts in an unsupervised manner, we specifically target the ‘polyp’ and ‘instrument’ concepts in this work. To identify channels that are likely to represent these concepts , we examine the corresponding CAVs obtained using TCAV. The top 9 to 12 channels receiving the highest average weights across the 20 example sets are selected for closer examination for each combination of concept (‘polyp’ and ‘instrument’) and class (‘abnormal’ and ‘normal’). Next, heatmaps are generated for each of the selected channels for each image class and concept. Only the heatmaps for the correctly predicted images in the representative test set are examined. The ResNetCanonizer from zennit is applied to extract the feature attributions from our ResNet152-based model ^33^. To avoid coarse-looking heatmaps, torchvision’s Gaussian blur with kernelsize 19 is used before saving the final heatmaps ^34^.

In addition to generating concept-dependent heatmaps for individual model predictions, we also use the CRP framework to examine prototypes that represent groups of similar model predictions. Unlike the heatmaps, these prototypes are not annotated but merely represent patterns in the data that the model has learned. With the PCX framework ^22^, we can visualize the predictions and prototypes in a two-dimensional plot: a prototype map. The map summarizes the concept-based explanations from a given dataset and model as prototypes, thereby explaining how the model interprets the dataset as a whole. These prototypes are derived in a fully unsupervised manner, and interpreted by manually studying the characteristic concepts. PCX can be used to quickly present the overall model behaviour as opposed to explaining a single model prediction at a time ^22^. In order to understand the characteristics of each prototype, the dominant concepts for the model, according to CRP, can be studied. To investigate how relevant a concept is for each prototype, we generate matrix-plots with concepts on the vertical axis and prototypes on the horizontal axis. Larger entry-values in the matrix indicate higher relevance for the given combination of concept and prototype .

### Expert feedback

To evaluate the model explanations among domain experts, images including polyps and/or instruments are manually selected from the representative test set, aiming to show diverse cases highlighting how the explanation methods perform in various settings . These images are not included in the training or validation datasets. The following explanations are generated: heatmaps by using GradCAM and CRP, CRP prototype visualizations with PCX, concept-prototype matrices and TCAV scores. A questionnaire including the resulting explanations along with a brief description of the study and nine questions was sent out to five medical doctors specialized in GI tract diseases and familiar with ML applications. The aim was to collect as much information about their impression of the explanations as possible. Participants were asked to share their thoughts on each of the explanation types separately, as well as to state which method(s) they preferred and why. They were also asked to consider whether they would prefer a single explanation method or a combination of several methods. Finally, because the prototype maps from the PCX framework and the concept-prototype matrices include unlabeled prototypes and concepts, we asked the medical experts if they could relate any of these to specific medical findings or diagnoses. The full questionnaire is available in D. All participants had provided written consent to contribute to the study.

## Supplementary Information

Supplementary Information.

## Data Availability

The datasets applied in the current work are openly accessible. Hyperkvasir, Kvasir-Instrument and Kvasir-SEG are available through the following links: https://datasets.simula.no/hyper-kvasir/, https://datasets.simula.no/kvasir-instrument/ and https://datasets.simula.no/kvasir-seg/. ImageNet images were accessed at the following web site: https://www.kaggle.com/competitions/imagenet-object-localization-challenge/data.

## Code availability

The code is made publicly available and can be accessed through the following link: https://github.com/AndreaStoraas/conceptXAI-GItract.
