# Supplementary material for: Exploring the clinical value of concept-based AI explanations in gastrointestinal disease detection
Source: Sci Rep. 2025 Aug 7;15:28860. doi: 10.1038/s41598-025-14408-y (PMC12332007; doi:10.1038/s41598-025-14408-y)
Supplement: Supplementary file 1 — Supplementary Information. [file 41598_2025_14408_MOESM1_ESM.pdf]

Supplementary Files

Title: Concept-Based Explainability of Deep Learning Models for Gastrointestinal Disease Detection

Authors: Andrea M. Storås, Maximilian Dreyer, Frederik Pahde,
Sebastian Lapuschkin, Wojciech Samek, Pål Halvorsen,
Thomas de Lange, Yuichi Mori, Alexander Hann,
Tyler M. Berzin, Sravanthi Parasa, Michael A. Riegler

Supplementary A PCX correlation matrices for concepts and prototypes

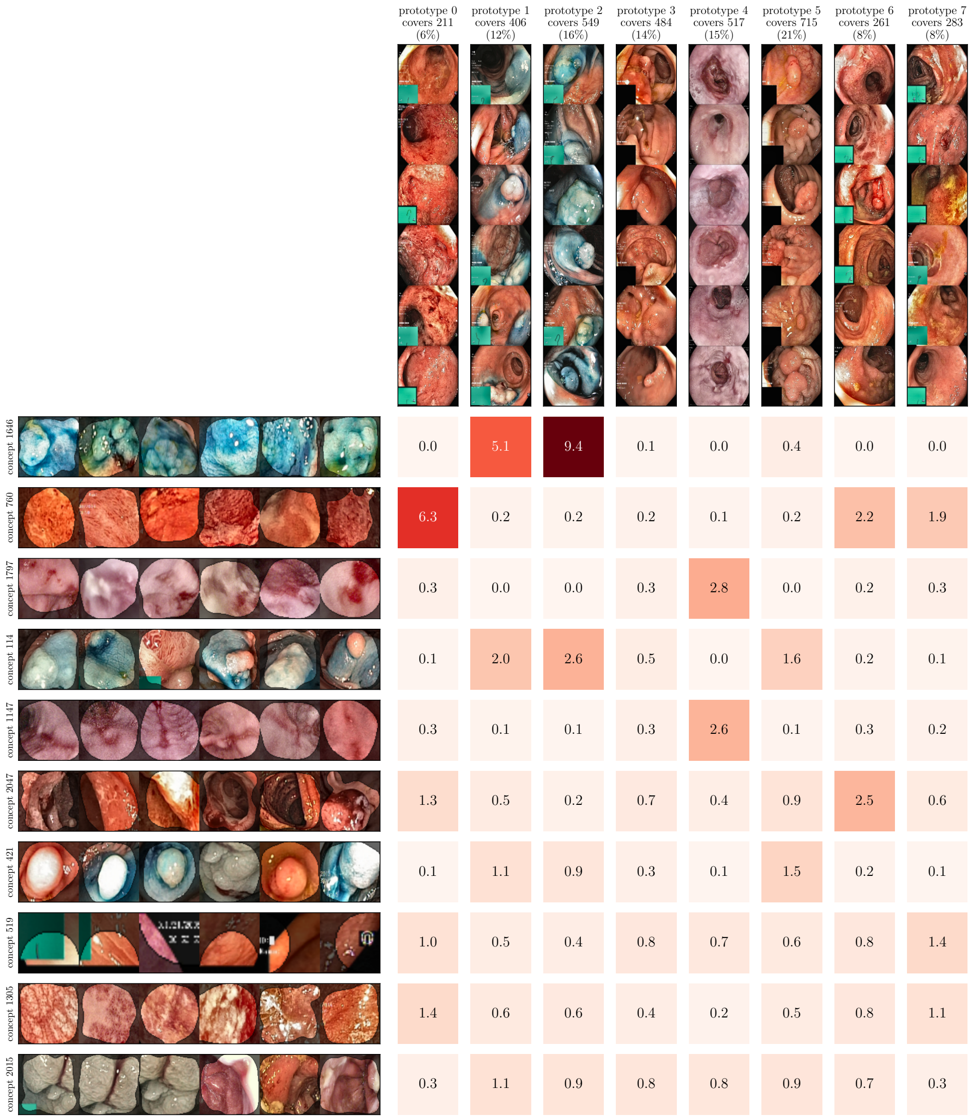

Fig. A.1: Correlation matrix for detected prototypes (horizontal axis) and concepts (vertical axis) for 'abnormal' images.

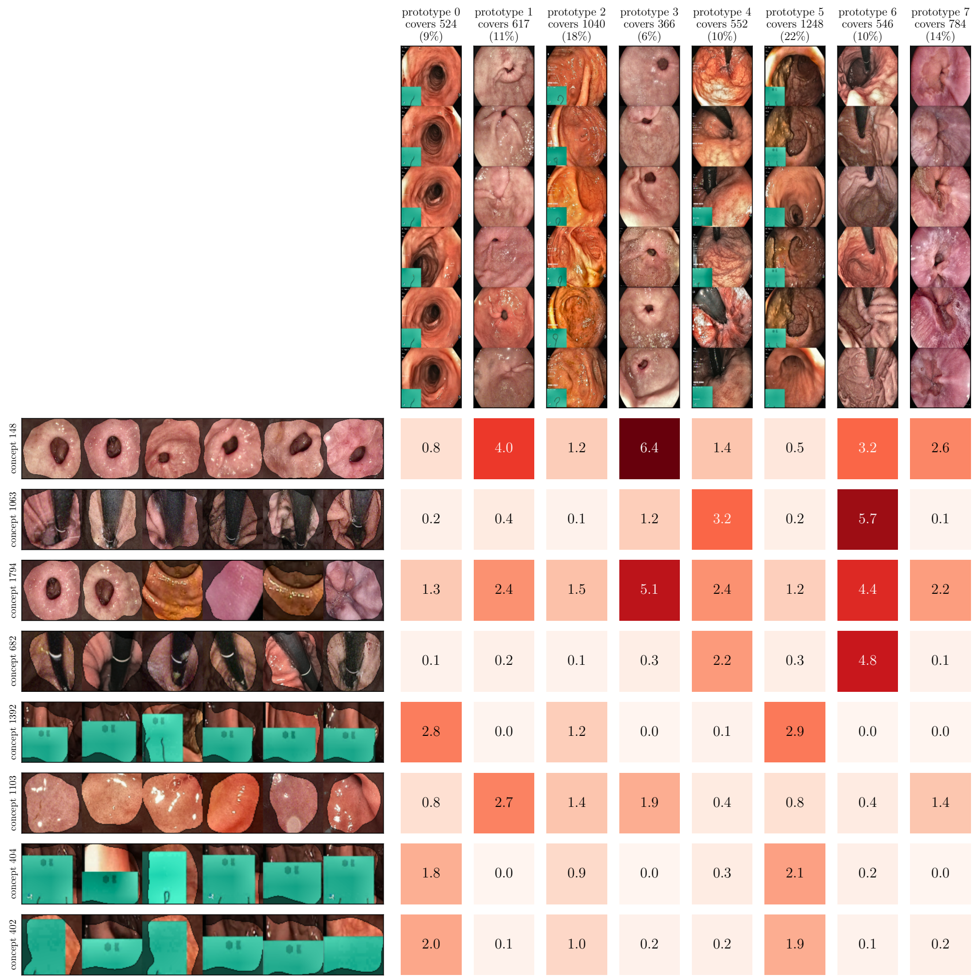

Fig. A.2: Correlation matrix for detected prototypes (horizontal axis) and concepts (vertical axis) for 'normal' images.

Supplementary B Testing for medical instrument

In this section, we describe the workflow for testing whether our model is sensitive towards the presence of a medical instrument (insertion tube). Therefore, we probe the model by inserting a cropped image of the insertion tube into images without the instrument, as proposed by Pahde et al. [7]. Specifically, we crop the artifacts out of artifact samples from 60 % of randomly chosen samples (652 samples with artifact) and insert it into the other 40 % of the remaining samples (3819 samples without artifact).

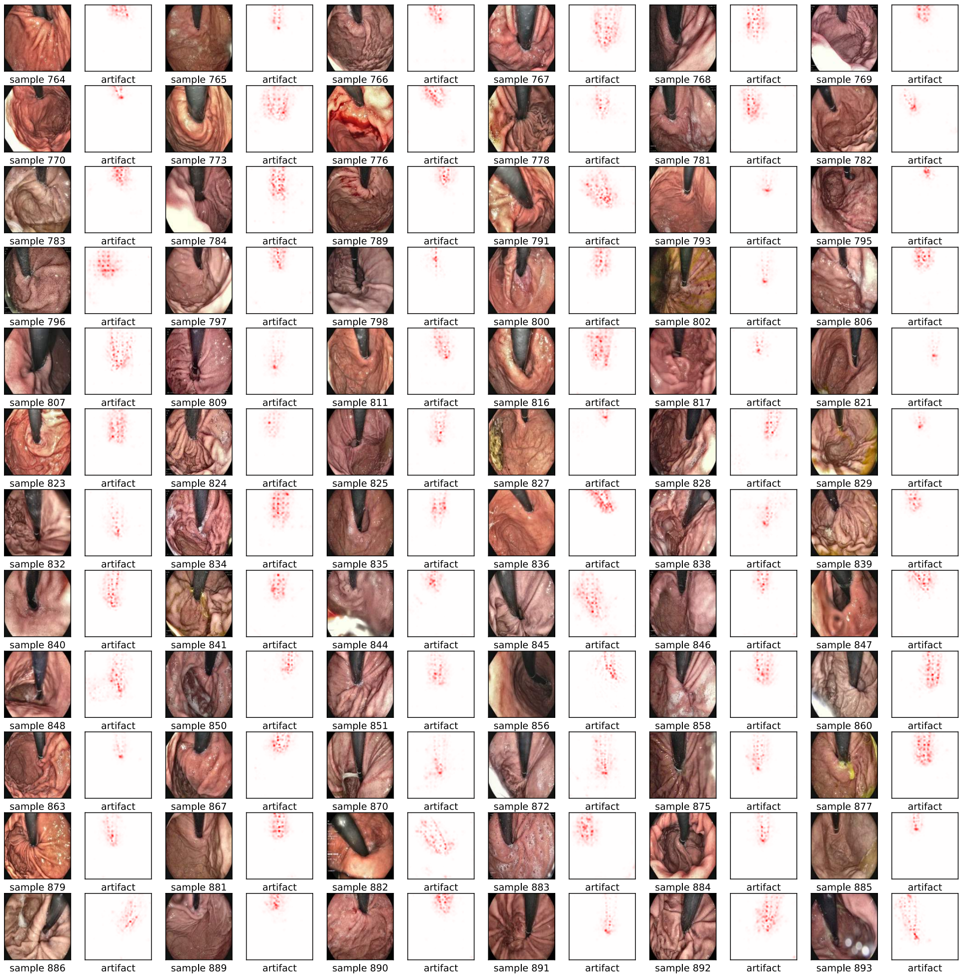

Fig. B.1: Localization maps for the medical instrument (insertion tube) used to test model sensitivity towards the artifact, by cropping the artifact out of artifact samples and inserting it in clean samples.

B.1 1) Labeling samples with artifact

In the first step, we leverage the tools described in [7] to find clusters using the SpRAy algorithm [30] based on clustering explanations (similar to PCX). We identify a cluster specifically dedicated to the medical instrument and can thus label all images containing and not containing the insertion tube.

B.2 2) Localizing the artifact

In the second step, we localize the instrument in the input images. Here, we compute a CAV (direction in latent space) based on the labels from the first step in the last

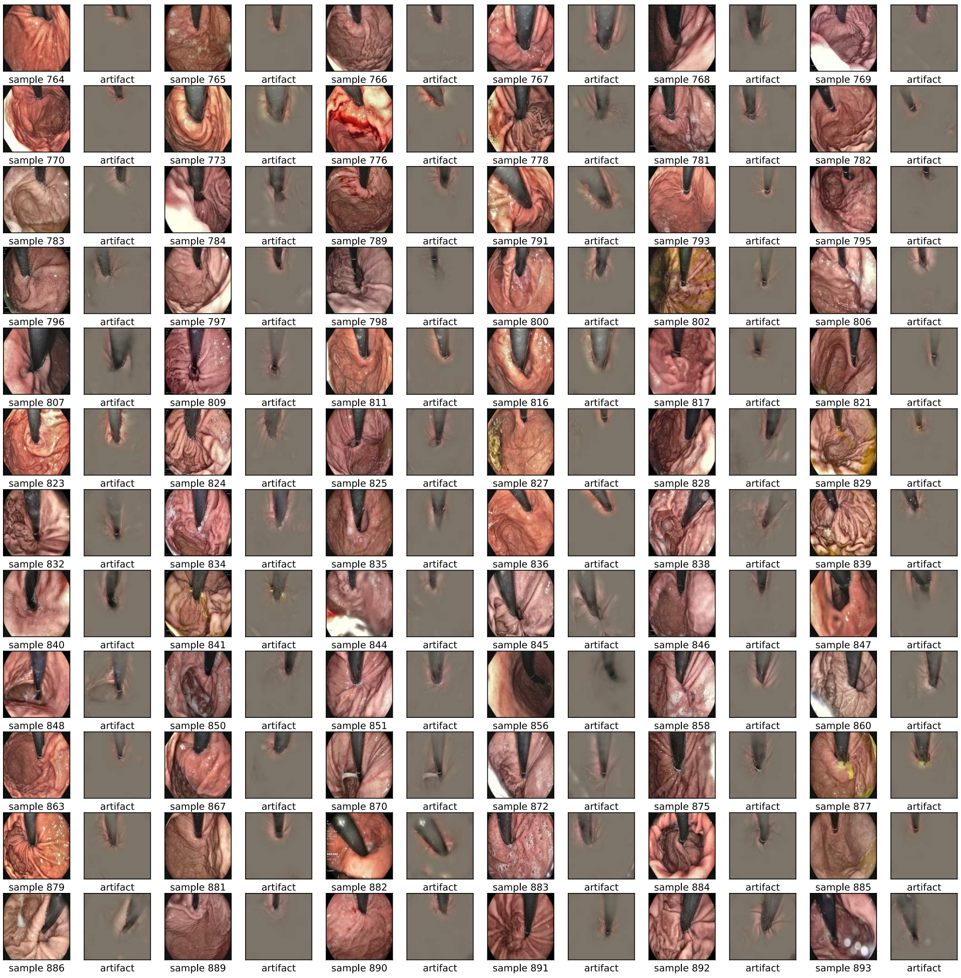

Fig. B.2: Cropping out the medical instrument (insertion tube) to test model sensitivity towards the artifact, by inserting the artifact in clean samples.

convolutional layer of the ResNet152-based model using a Support Vector Machine. This can also be seen as identifying neurons that activate specifically for the samples where the artifact is present. Afterward, we can explain the dot product between latent activations and this direction, resulting in a heatmap that points towards the artifact, as shown in Figure B.1.

B.3 3) Cropping and inserting the artifact

In the third step, we crop out the artifact after preprocessing the localizations by applying Gaussian blur (kernel size of 41, sigma of 7.0), taking all values to the power of 0.6 and normalization to $[0, 1]$. The cropped artifacts are shown in Figure B.2.

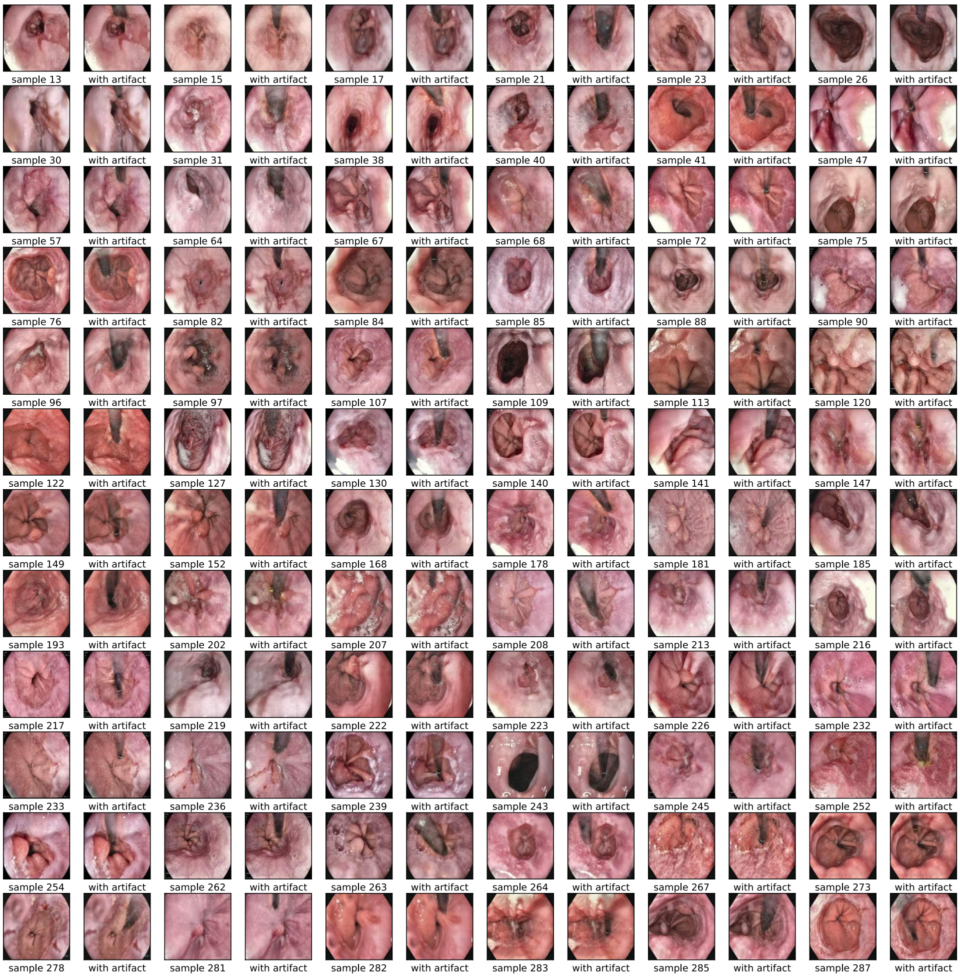

Fig. B.3: Testing model sensitivity towards the medical instrument (insertion tube), by cropping the artifact out of artifact samples and inserting it in clean samples. Shown are test samples with and without inserted artifact.

Notably, the masks used for cropping are not binary, leading to smooth crops and insertions. Examples for samples with inserted artifacts are shown in Figure B.3.

When inserting the artifact into the 3819 clean samples from the hold-out set, the accuracy for the abnormal class drops from 0.943 to 0.783, and it increases for the normal class from 0.949 to 0.967. These results confirm the findings by CRP, indicating that the insertion tube is used by the model as a spurious pattern to indicate normality.

Supplementary C Concept relevance propagation explanations for images with instruments

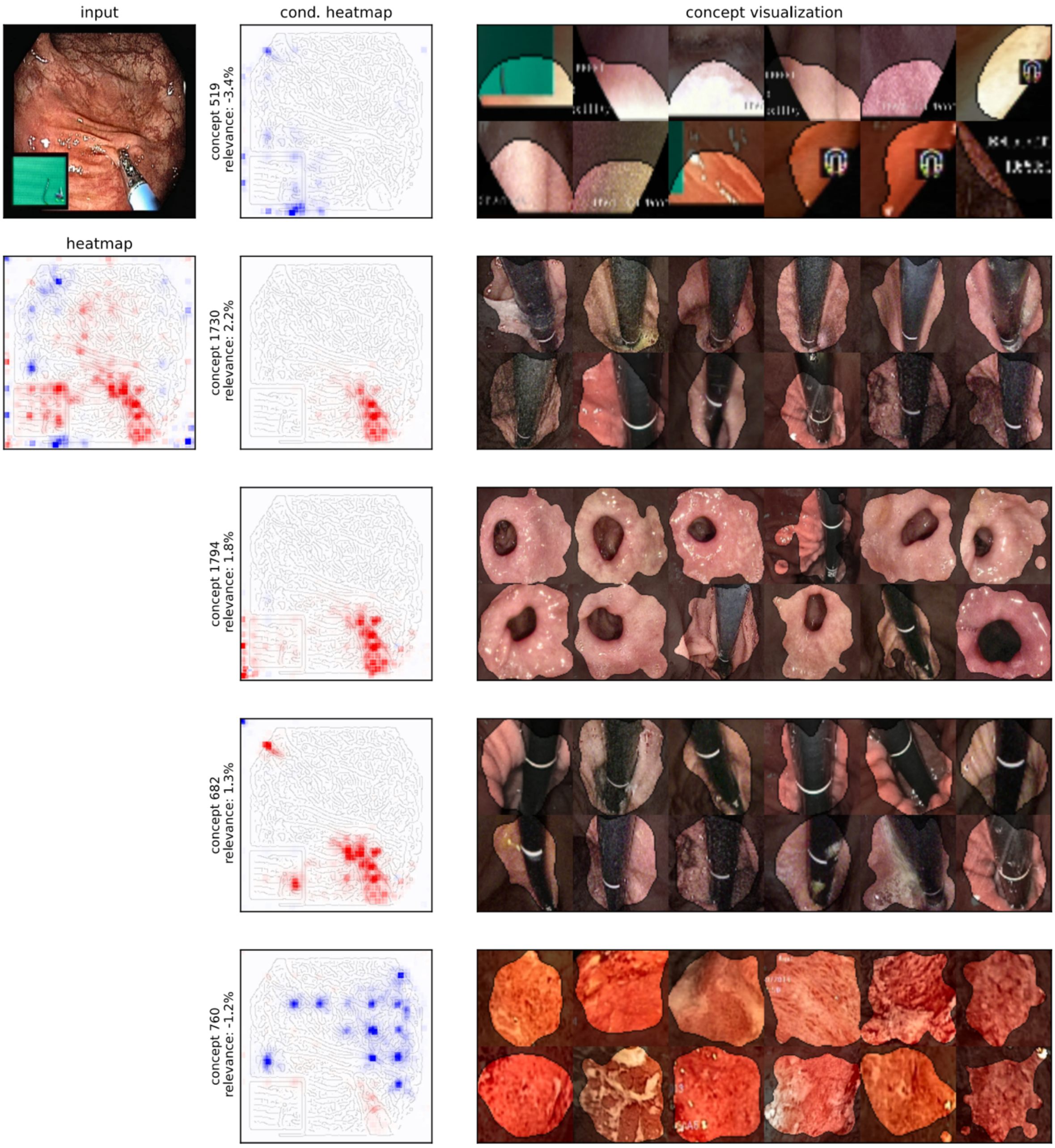

Fig. C.1: Image containing an instrument (upper left) and corresponding concept relevance propagation heatmaps and representative images for the most relevant concepts. The image was predicted as 'normal'. Concept 519 (upper row) represents black edges and text and is relevant for the 'abnormal' class. Concepts 1730, 1794 and 682 (three middle rows) represent black instruments and are relevant for the 'normal' class. Concept 760 (bottom row) is relevant for the 'abnormal' class.

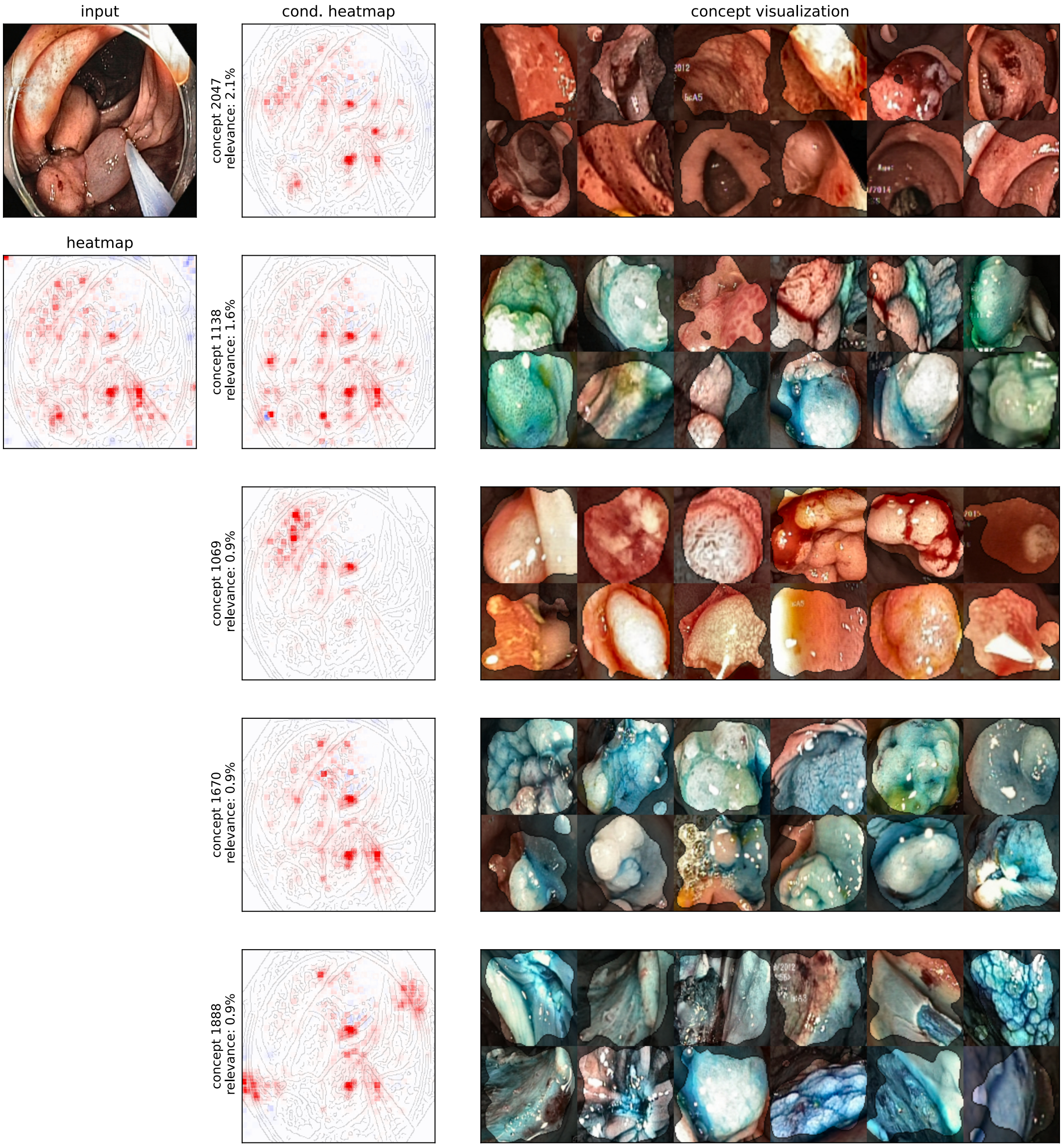

Fig. C.2: Image containing a bright white instrument (upper left) and corresponding concept relevance propagation heatmaps and representative images for the most relevant concepts. The image was predicted as ‘abnormal’. None of the most relevant concepts represent instruments.

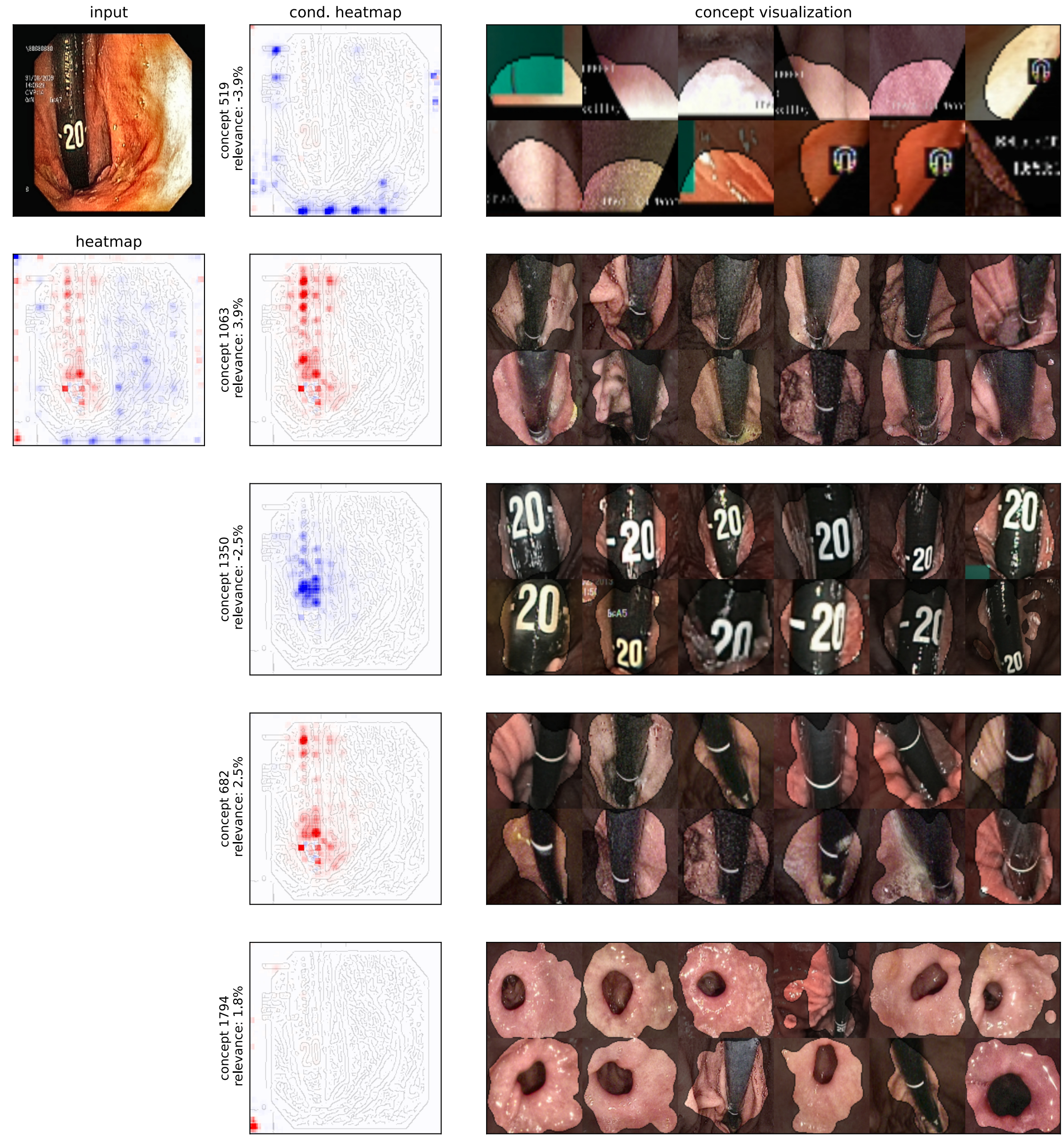

Fig. C.3: Image containing an instrument (upper left) and corresponding concept relevance propagation heatmaps and representative images for the most relevant concepts. The image was predicted as 'normal'. Concept 519 (upper row) represents black edges and text and is relevant for the 'abnormal' class. Concepts 1063, 682 and 1794 (second upper row and two bottom rows) represent black instruments and are relevant for the 'normal' class. Concept 1350 (middle row) represents black instruments where the number 20 is visible and is relevant for the 'abnormal' class.

Supplementary D Questionnaire for expert feedback

Doctor feedback on model explanations for the gastrointestinal tract

The doctor feedback task

This document includes several types of model explanations that aim to provide insights into how a deep neural network (DNN) analyses images of the gastrointestinal (GI) tract. We kindly ask you to provide feedback for each group of explanations. Specifically, we want you to reply to the following questions:

1. What are your thoughts about the GradCAM heatmaps for explaining model predictions?
2. What are your thoughts about the CRP heatmaps for explaining model predictions?
3. What are your thoughts about the CRP prototype-concept matrices for explaining overall model behaviour?
4. Regarding the CRP prototypes: What would you name the different prototypes? Do any of the prototypes represent a particular type of medical finding or diagnosis?
5. What are your thoughts about the UMAP embeddings for explaining overall model behaviour?
6. What are your thoughts about TCAV scores for explaining overall model behaviour?
7. Which explanation type(s) do you prefer and why?
8. If you had to pick one explanation method, which one would it be?
9. Can a combination of explanation methods be better than one single method? If so, which of them would you combine?

Below follows some information about the model to be explained and the various explanation types. The explanations are provided at the end of the document.

The deep neural network

In this project, we train a DNN to identify abnormal images of the GI, i.e., images representing a disease in the GI tract. The model is trained on images from the labeled subset of the Hyperkvasir dataset. Images representing barretts, esophagitis, polyps, ulcerative colitis or hemorrohoids are defined as “disease” (class 1), while images of anatomical landmarks in the GI tract (pylorus, retroflex-stomach, z-line, cecum, ileum and retroflex-rectum) as well as quality of mucosal view are defined as “no disease” (class 0).In other words, we train a binary classifier to predict whether an image represents “disease” (class 1) or “no disease” (class 0).

Model explanations

The resulting model is explained using traditional heatmaps from GradCAM and by applying two different concept-based methods: testing with concept activation vectors (TCAV) and concept relevance propagation (CRP). A concept can be defined as a topic or theme, such as “stripes” or “dots”. Explanations based on concepts have been claimed to be more human-friendly compared to other explanation types. An advantage with concept-based methods is that the concepts can be tailored to the use case which facilitates relevant explanations.
For this project, where we analyze images from the GI tract, we explore “polyps” and “instruments” as concepts. Since polyps are included in some (but not all) images representing the disease class, we expect the model to pay attention to this concept when detecting disease. Instruments are present in images from both classes and are expected to be less important for the model when learning to separate images with and without disease.

Heatmaps

The concept-based method called CRP method produces heatmaps highlighting concepts of interest in the input images. As opposed to GradCAM heatmaps, the highlighted areas in the CRP heatmaps concern a specific individual concept, as utilized by the model during inference. This enables us to inspect not only *where* in the image the model focuses on, but also *what* those areas represent. The CRP method assumes that a specific node or channel in the DNN represents a concept. Consequently, we map model channels to specific concepts, such as “polyp” and “instrument”. Red areas in the CRP heatmaps mean positive relevance. In other words, the channel for the given concept is positive for the specific model prediction. Blue areas in the CRP heatmaps mean negative relevance, and the channel for the given concept is negative for the specific model prediction.

GradCAM heatmaps do not rely on any concepts and simply show which areas in the image that are important for the DNN based on the gradients during backpropagation. Areas that are of highest importance are shown with bright yellow and red colors in the GradCAM heatmaps, while the least important areas are dark blue.

For GradCAM, we obtain one heatmap for each image. For CRP, we include one heatmap per concept, i.e., for one single image we include one CRP heatmap for the “polyp” concept and one CRP heatmap for the “instrument” concept. We include heatmaps for both concepts even if the original image does not contain both polyps and instruments.

Prototypes and UMAP embeddings

Studying single explanations is usually unfeasible for large datasets. In order to ease the process of understanding what the model has learned on the whole dataset, we summarize groups of similar predictions via a few (here eight) prototypes.

With the UMAP (Uniform Manifold Approximation and Projection) algorithm, we can visualize the predictions and prototypes in a two-dimensional plot (“map”). Here, points that are close, correspond to a similar prediction (and explanation).

In order to understand the characteristics of each prototype, we can study the dominant concepts (according to CRP) for the model. In form of a matrix-plot, we can see how relevant (matrix entry value) a concept (vertical axis, with concept visualization) is for all prototypes.

TCAV scores

TCAV scores, which are obtained by using the concept-based TCAV method, represent the fraction of images, e.g., the fraction of all class 1 images, for which the model reacts to in the direction of a vector representing a specific concept. High TCAV scores for a concept indicate that this concept is important for the model when making predictions on the group of images.

Heatmaps for class 1 (disease) image predictions

Original images

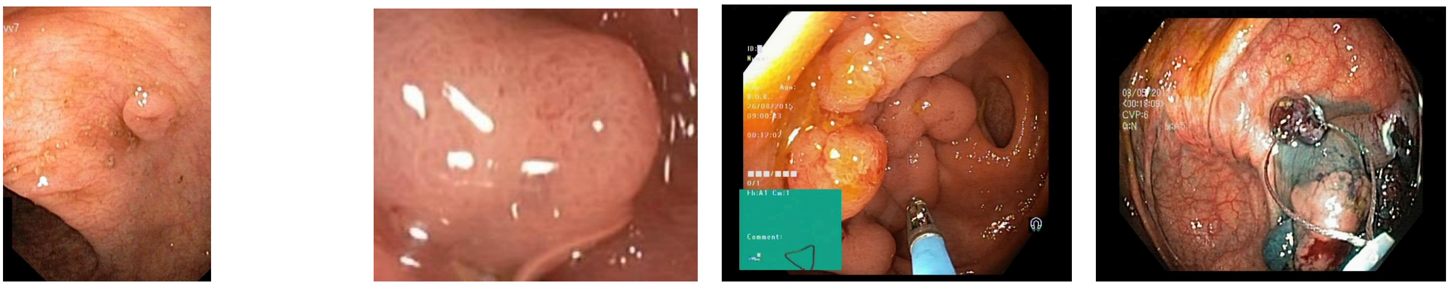

GradCAM heatmaps

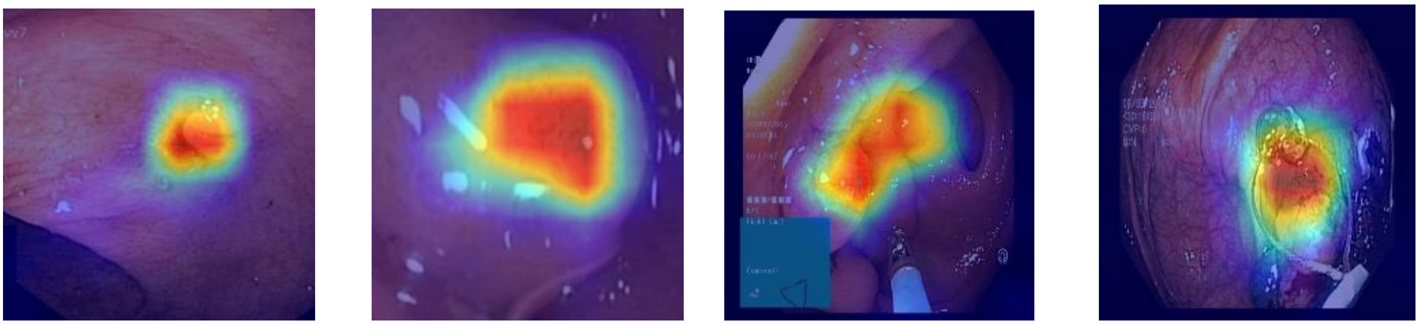

CRP heatmaps and contribution to model prediction: polyp concept

1.41% 1.23% 1.90% 0.52%

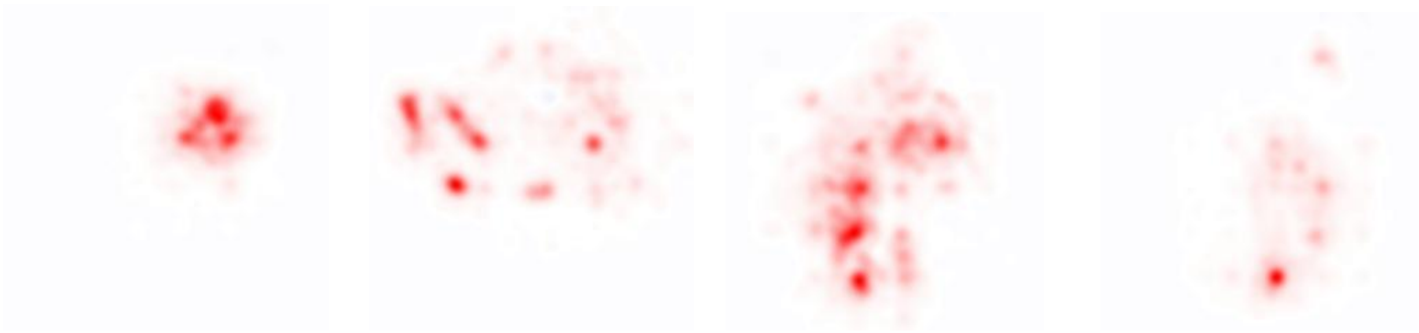

CRP heatmaps and contribution to model prediction: instrument concept

-0.01% -0.04% -1.27% -0.17%

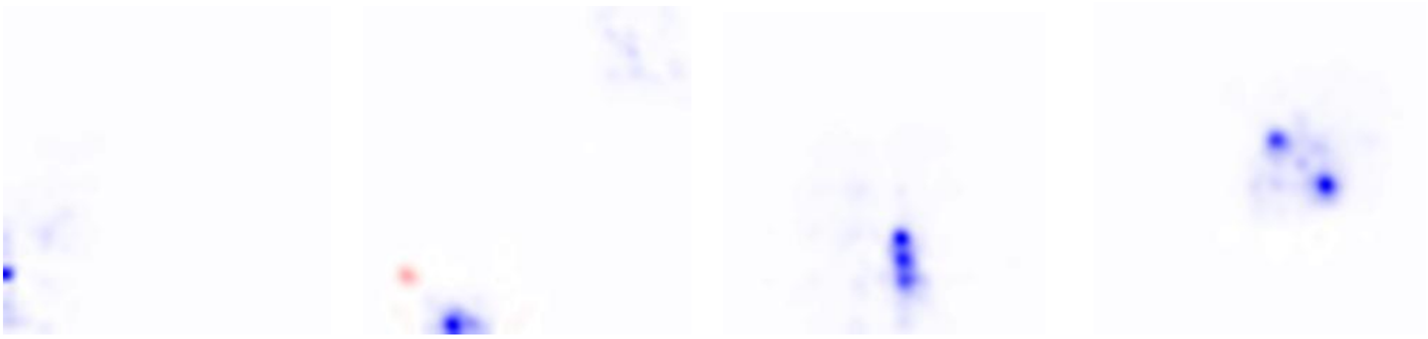

Heatmaps for class 0 (no disease) image predictions

Original images

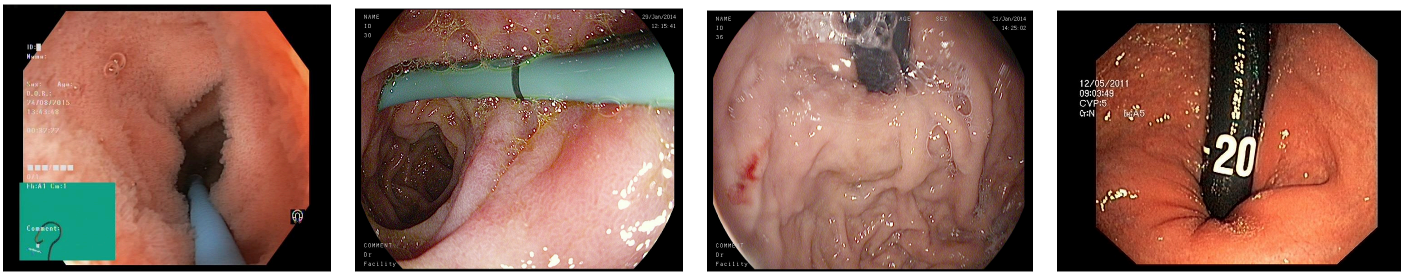

GradCAM heatmaps

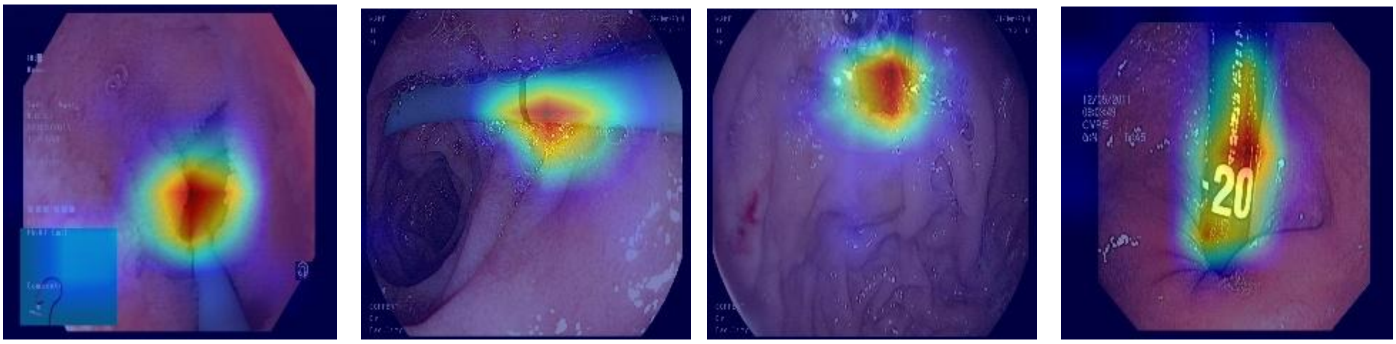

CRP heatmaps and contribution to model prediction: polyp concept

-0.04% -0.37% -0.004% -0.16%

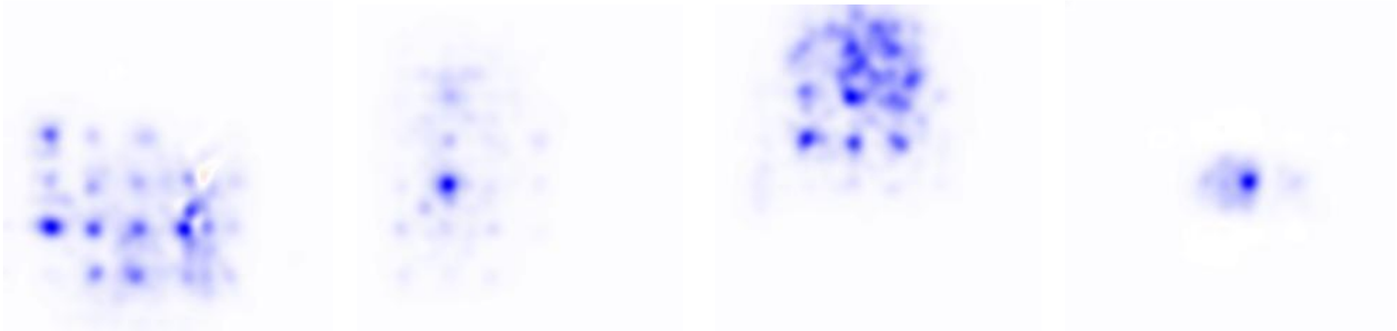

CRP heatmaps and contribution to model prediction: instrument concept

2.67% 2.83% 2.09% 3.13%

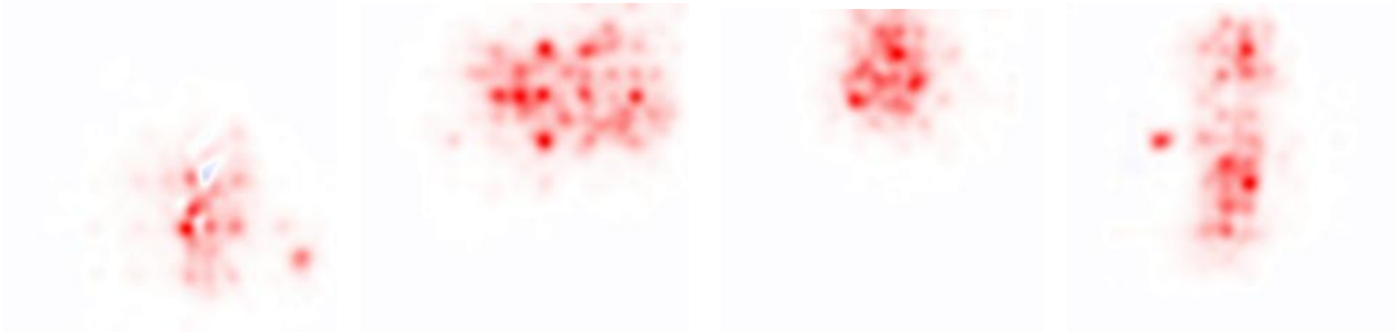

Prototype-concept matrix for class 1 (disease) images

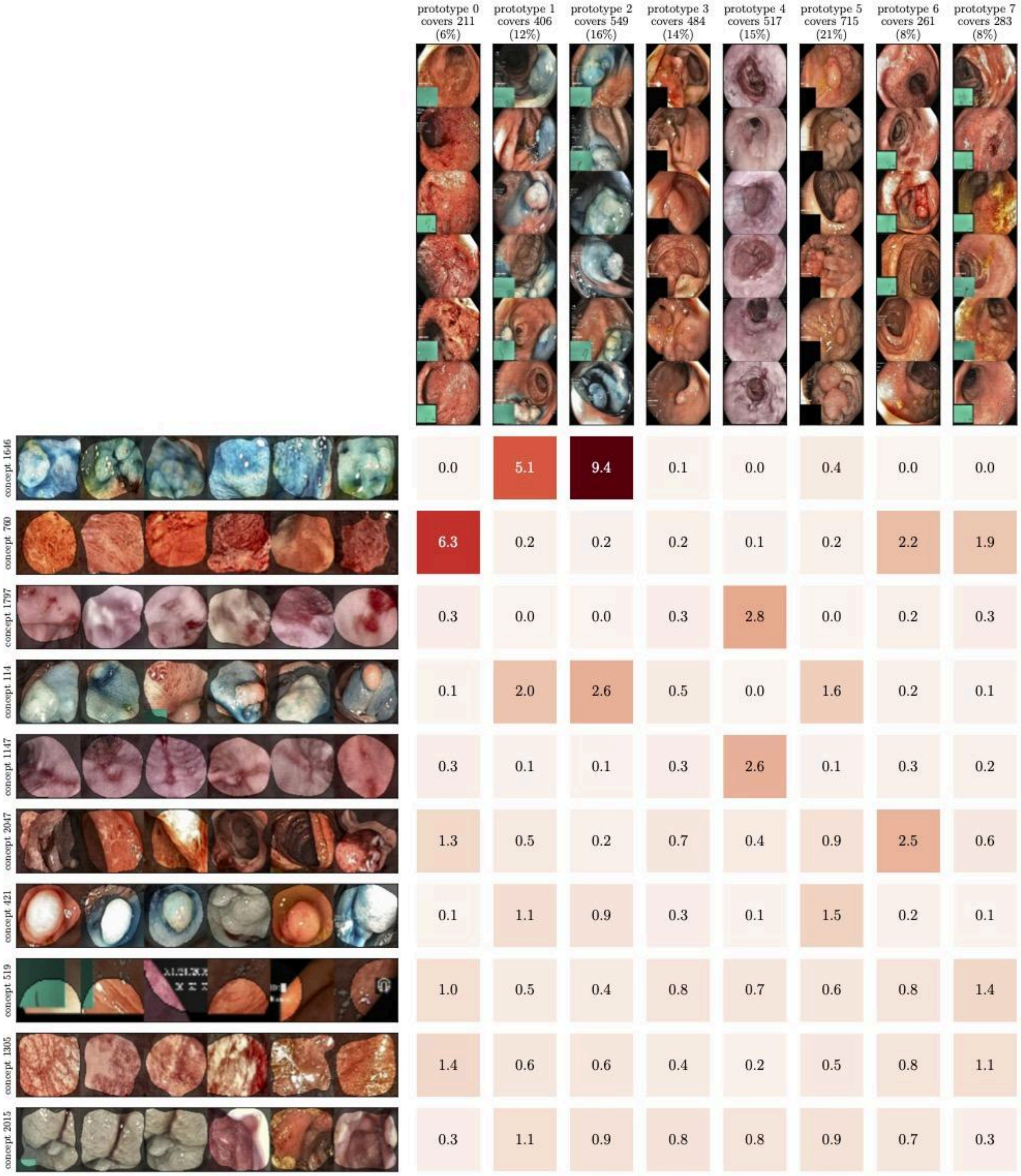

Class 1 prototypes with their most representative images are shown as columns, while concepts with their most representative images are shown as rows.

Prototype-concept matrix for class 0 (no disease) images

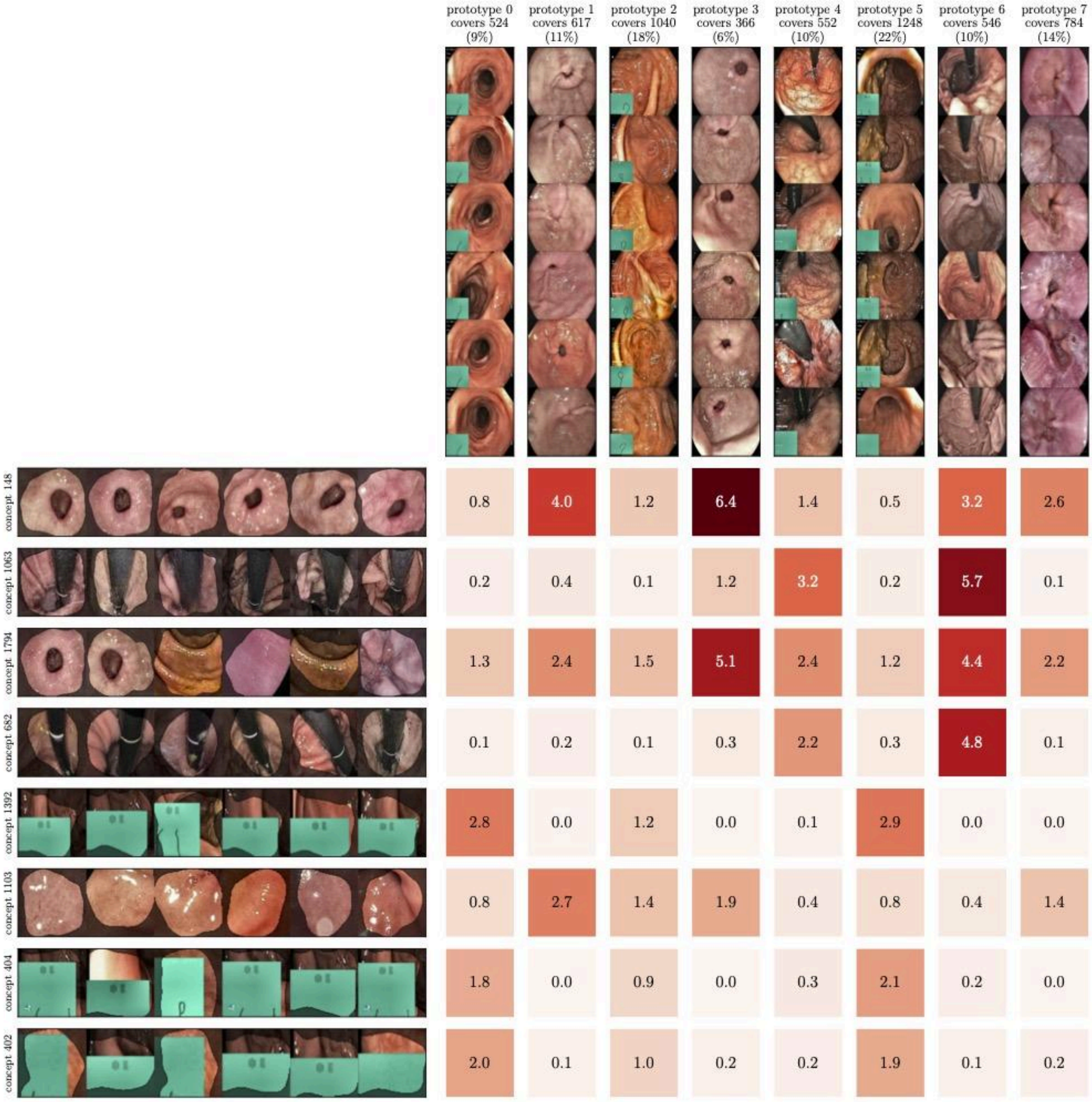

Class 0 prototypes with their most representative images are shown as columns, while concepts with their most representative images are shown as rows.

UMAP embeddings for class 1 (disease) images

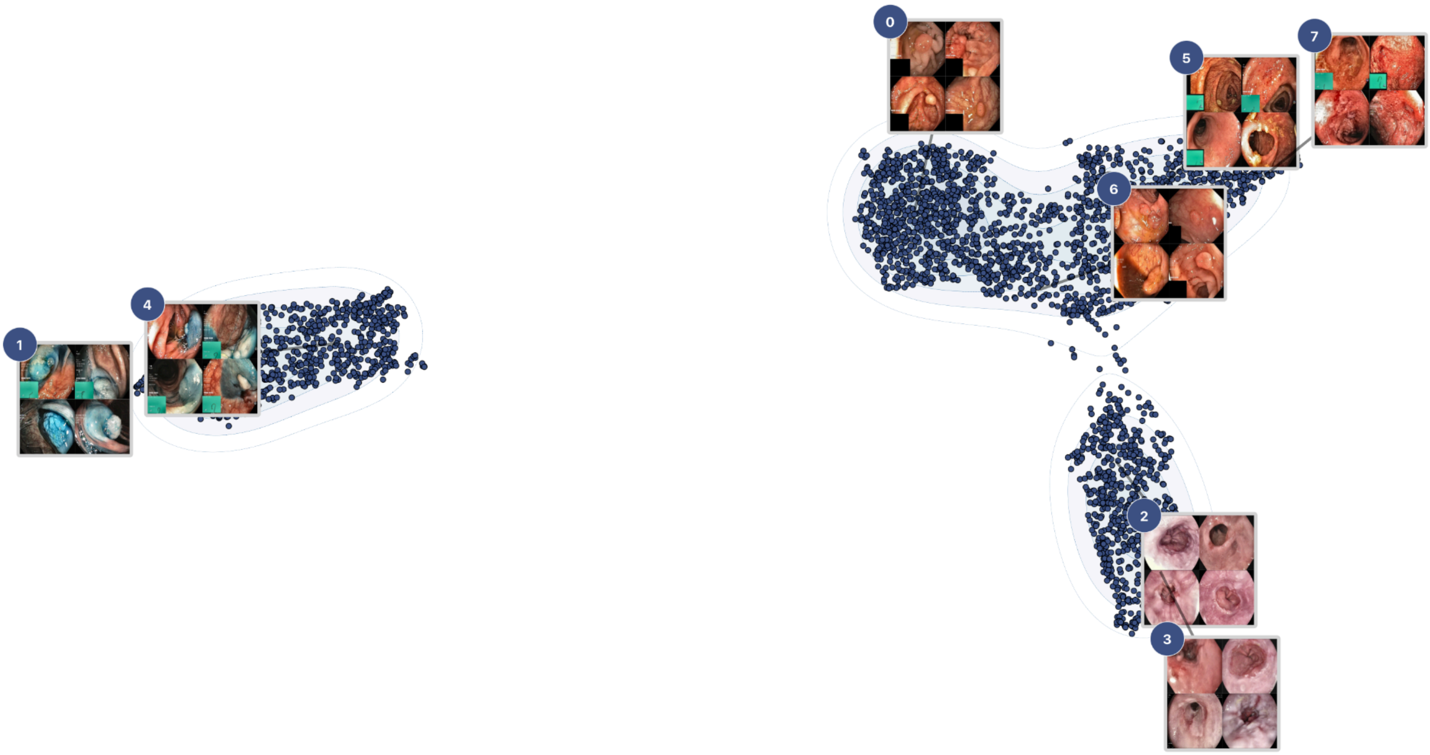

UMAP embeddings for class 0 (no disease) images

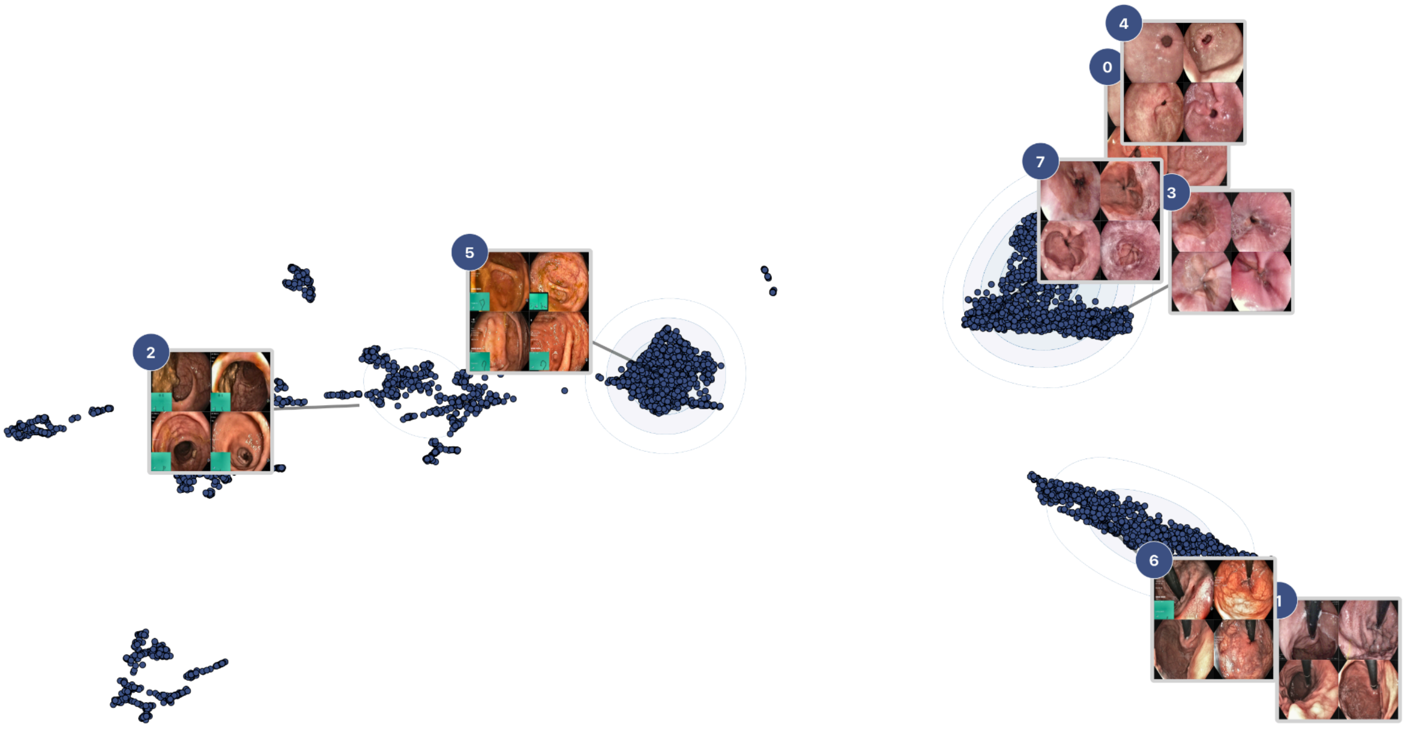

TCAV scores for each combination of image class and concept type

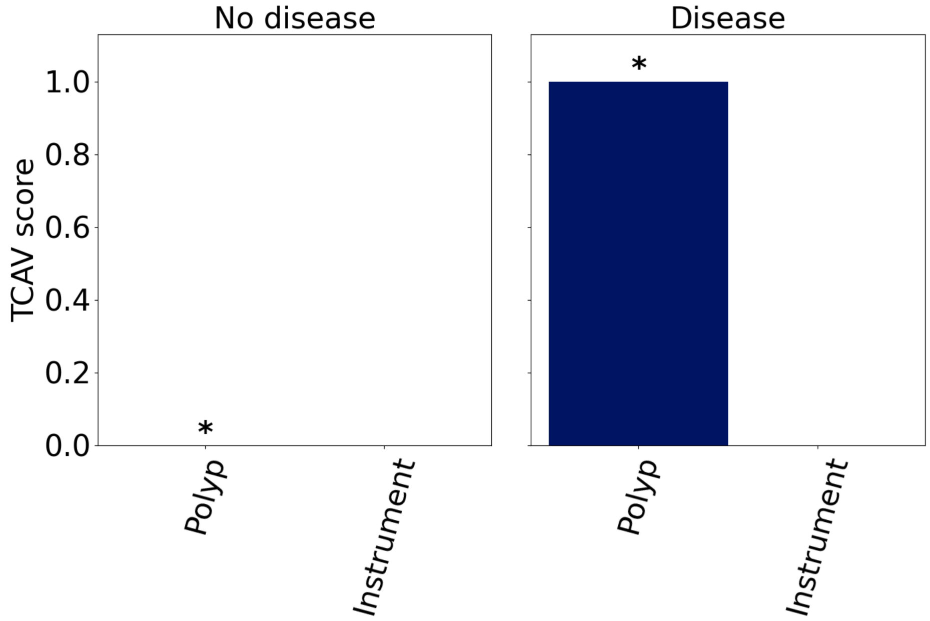

The mean TCAV scores across 20 pairs of positive and negative concept example sets are plotted for both classes (no disease and disease). Significant concepts are marked with *. Insignificant concepts have their scores set to 0. The highest possible score is 1, indicating that for all the example sets, the model is always affected in the direction of the concept when making predictions on the images. The “No disease” plot to the left shows the TCAV scores for the class 0 images, while the “Disease” plot to the right shows the corresponding scores for the class 1 images.
